# Editorial: The role of nutrition in mitigating depression: mechanisms, interventions, and outcomes

**DOI:** 10.3389/fnut.2026.1754815

**Published:** 2026-03-16

**Authors:** Roberta Zupo, Fabio Castellana, Giuseppe Colacicco, Rodolfo Sardone, Feliciana Catino, Maria Lisa Clodoveo

**Affiliations:** 1 Department of Interdisciplinary Medicine (DIM), University of Bari Aldo Moro, Bari, Italy; 2 Department of Translational Biomedicine and Neuroscience (DiBrain), University of Bari Aldo Moro, Bari, Italy; 3 Urban Health Center-Local Health Authority of Taranto and the Municipality of Taranto, Taranto, Italy

**Keywords:** depression, diet, food, nutrition, public health

## 1 Introduction

The intricate relationship between diet and mental health has gained substantial scientific attention over the past decade, with particular focus on how nutritional factors may influence the onset, course, and severity of depressive symptoms. Recent evidence, including a systematic review demonstrating a non-linear dose–response relationship between the Dietary Inflammatory Index (DII) and depression risk (1), reinforces the role of diet as a key determinant of mental health.

The Research Topic “*The role of nutrition in mitigating depression: mechanisms, interventions, and outcomes*” brings together six contributions—original research and review papers—that collectively advance our understanding of how dietary patterns, inflammation, metabolic dysregulation, and the gut microbiome converge in shaping depression risk and resilience. This Editorial aims to synthesize these findings, highlight their complementarity, and outline future directions for research in nutritional psychiatry.

## 2 Emerging evidence across biological and behavioral domains

A cornerstone of this Research Topic is the review *Gut microbiota, nutrients, and depression* (Qiao et al.), which synthesizes current insights into the bidirectional communication along the gut–brain axis. The authors emphasize how fermentable fibers, polyphenols, fatty acids, and micronutrients modulate microbial diversity, short-chain fatty acid production, and neuroactive metabolite signaling. These mechanisms influence neuroinflammation, neurotransmission, and stress-response circuits, reinforcing the notion that diet is a modifiable biological regulator of mental health.

In parallel, the original study *Association between the dietary inflammatory index and depressive symptoms in adults with cardiovascular–kidney–metabolic syndrome* (Tian et al.) provides population-level evidence linking higher DII scores to increased depressive symptoms, with metabolic syndrome acting as a partial mediator. These results align with those of Yu et al. (1), who identified a non-linear association between inflammatory dietary potential and depression.

Additional contributions expand this landscape, examining nutrient status, overall dietary quality, lifestyle behaviors, and nutrition-related health outcomes. These include studies on plant-based diets and sleep as mediators of depression in older adults with heart disease (Yu et al.), vitamin deficiencies as contributors to late-life depression (Gao et al.), the relationship between food preferences and depressive symptoms among undergraduate medical students (Sedgi et al.), and the association between vitamin D deficiency and major depression in individuals with chronic kidney disease (Chen et al.).

Despite their methodological diversity, these studies collectively demonstrate that nutrition influences depression through a constellation of interconnected systems—metabolic, inflammatory, microbial, endocrine, and behavioral.

## 3 A unified conceptual framework

### 3.1 Nutrition acts as a multisystem regulator

Across studies, dietary patterns modulate multiple biological systems simultaneously. Anti-inflammatory, fiber-rich, and nutrient-dense diets support metabolic health, reduce systemic inflammation, and promote favorable gut microbial profiles—mechanisms consistently supported across the Research Topic (1) (Qiao et al.; Tian et al.; Yu et al.; Gao et al.; Sedgi et al.; Chen et al.).

### 3.2 Individual context matters

Associations between nutrition and depression emerge more strongly in individuals with cardiometabolic comorbidities, chronic inflammation, or renal impairment. Evidence from Tian et al., as well as findings related to micronutrient deficiencies (Gao et al.) and vitamin D status (Chen et al.), highlights the importance of personalized nutrition strategies.

### 3.3 No single “anti-depressant diet” exists

Across contributions, benefits derive from overall dietary quality rather than rigid dietary prescriptions. Whole-food diets rich in fiber, antioxidants, and anti-inflammatory components consistently demonstrate protective associations, while highly processed, pro-inflammatory foods are associated with increased depression risk (1) (Qiao et al.; Tian et al.; Yu et al.; Gao et al.; Sedgi et al.).

### 3.4 Public health implications

This body of evidence underscores the importance of integrating nutritional strategies into mental health prevention and treatment. Policy actions to improve access to healthy foods, reduce dietary inequalities, and enhance food environments could meaningfully reduce population-level depression risk.

## 4 Gaps and future research directions

Despite substantial progress, important gaps remain. Most included studies are observational, limiting causal inference. Heterogeneity in dietary assessment tools, inflammatory indices, and depression measures complicates interpretation. Future research priorities include:

- longitudinal and interventional studies, including well-powered randomized controlled trials;
- integration of multimodal biomarkers (metagenomics, inflammatory markers, metabolomics, neuroimaging);
- precision-nutrition approaches informed by metabolic, microbial, and sociodemographic features;
- explicit consideration of social determinants such as food affordability, cultural dietary practices, and socioeconomic inequalities.

## 5 Conclusion

Together, the contributions in this Research Topic—and complementary meta-analytic findings (1)—emphasize that nutrition represents a powerful, modifiable determinant of depression risk and symptom trajectory. Dietary patterns interact with metabolic health, inflammatory pathways, and the gut microbiome to shape mental wellbeing.

This integrated perspective informs clinical practice, public health policy, and future research priorities. We hope this Research Topic will stimulate further multidisciplinary collaboration and inspire innovative nutrition-based approaches to promoting mental health worldwide.
